# The Effect of Different Antibiotic Regimens on Bacterial Resistance: A Systematic Review

**DOI:** 10.3390/antibiotics9010022

**Published:** 2020-01-08

**Authors:** Romeo Patini, Gilda Mangino, Leonardo Martellacci, Gianluca Quaranta, Luca Masucci, Patrizia Gallenzi

**Affiliations:** 1 Institute of Dentistry and Maxillofacial Surgery, Fondazione Policlinico Universitario A. Gemelli IRCCS, Università Cattolica del Sacro Cuore, 00168 Rome, Italy; gildamangino@gmail.com (G.M.); lmartellacci@virgilio.it (L.M.); patrizia.gallenzi@unicatt.it (P.G.); 2 Institute of Microbiology and Virology, Fondazione Policlinico Universitario A. Gemelli IRCCS, Università Cattolica del Sacro Cuore, 00168 Rome, Italy; gquaranta88@gmail.com (G.Q.); luca.masucci@unicatt.it (L.M.)

**Keywords:** antibiotic, bacterial resistance, systematic review, prophylaxis

## Abstract

Background and objectives: Infections caused by resistant bacteria are a growing public health problem that is linked to many different causes, among them the antibiotics’ incorrect use plays an important role. According to the World Health Organization (WHO) the most dangerous behaviors are the early interruption of antibiotic therapy and the use of molecules without appropriate prescription. The authors conducted a systematic review to assess if antibiotic prescription with different regimens is connected to the onset of bacterial resistance. Methods: The authors performed an electronic and manual literature search on four databases (Web of Science, Scopus, PubMed, and Cochrane Register of Controlled Trials) from their inception to 15 June 2019. The date of the last search was 27 November 2019. Any article comparing cultural or genic analysis of resistance in patients that took antibiotics with at least two different regimens was included. No language restrictions were applied. Risk of bias for randomized controlled trials (RCTs) was assessed using the Cochrane collaboration’s tool whereas case-control and cohort studies were evaluated through the Newcastle–Ottawa scale. Results: The initial search resulted in a total of 1744 titles. After careful evaluation of all results, only three studies satisfied the outcome of the present review. From the qualitative analysis of data, it emerges that even if antibiotics are administered for a shorter period than the conventional one the species that inhabit the oral cavity can adapt quickly and express genes of antibiotic resistance. Additional evidence from this analysis is that not only does the proportion of resistant bacteria increase in the oral cavity, but also in more distant districts such as the intestine. Conclusions: Despite the great number of studies retrieved by electronic databases only few studies investigated the target of this review. The reason for this evidence is that it is not ethical to investigate and compare different antibiotic regimens, shorter or longer than the appropriate one. This evidence is applicable both to prophylactic administrations and to those aimed at treating infections. Besides this, the WHO affirms that, in the absence of infective complications, the prescription of antibiotic after every type of surgical intervention cannot be admitted and that studies dealing with antibiotic regimens that do not comply with drug’s pharmacodynamics characteristics cannot be ethically admitted. PROSPERO acknowledgement of receipt [149149].

## 1. Introduction

Bacterial resistance is the capability of the bacteria to multiply in the presence of drug concentrations that are mostly inhibitors of the same species or equal to the maximum achievable concentration during the therapeutic use. The rising of resistant bacteria is a growing public health problem that is mainly linked to antibiotics’ incorrect use. One of the connected problems consists of the increasing complexity of medical treatment and the growing amount of costs that is estimated as $30 billion annually [1].

Other important issues are the over or incorrect prescription, patients not finishing antibiotic treatments, the overuse in livestock and fish farming, and the lack of hygiene and poor sanitation and infection control in hospitals and clinics [1]. There are two types of resistances: intrinsic and acquired [2].

Previous studies have already demonstrated that patients’ noncompliance towards antibiotic prescription is an emerging issue and that it is mainly linked to misunderstanding of the treatment [3,4,5,6].

Evidence has been already highlighted, in form of a call to action, against the indiscriminate use of antibiotics for animal agriculture and fish farming [7] and regarding the strict necessity to manage healthcare associated infections that remain one of the biggest causes of death in most countries [8].

Strategies to cope with increasing bacterial resistance include accurate knowledge of the microbial component that feeds infections [9,10] and the use of biological agents for their control [11].

In medicine and dentistry, the prescription of antibiotics in recent decades has been the subject of debate since many authors presented results of studies in which a reduction of prescription duration resulted in substantial equal effects in terms of infection resolution and postoperative adverse effects onset [12,13,14,15,16,17]. Since the World Health Organization (WHO) clearly reported that incorrect or over-prescription of antibiotics is one of the main causes of the development of resistance and since the abovementioned “short course therapies” are in contrast with pharmacological appropriate posology, it is plausible to hypothesize that these prescriptions have effects on the onset of bacterial resistance. For this reason, the purpose of the present review was to evaluate the existing literature about the role of different antibiotic regimens administered for therapeutic and prophylactic purposes in provoking bacterial resistance [1].

## 2. Results

### 2.1. Results of the Search

**Figure 1:**
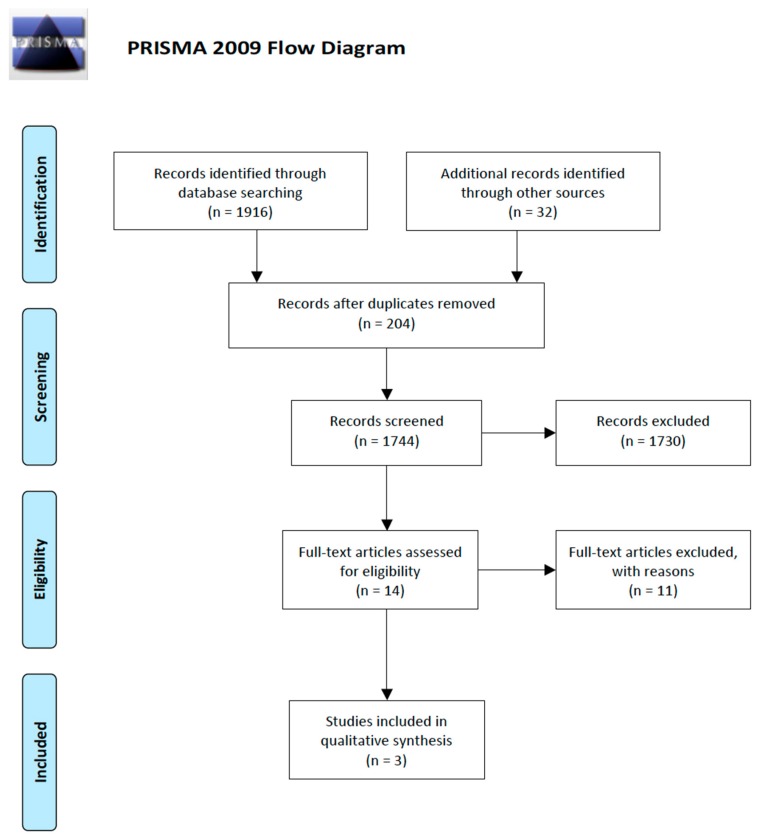
Flow chart of the screening process.

When the electronic and the manual search were completed 1948 titles matched the inclusion criteria. The elimination of duplicate results led to 1744 titles of which 1730 were removed analyzing their title and abstract. According to this process three randomized controlled trials (RCTs) were selected and included in this review (Figure 1).

### 2.2. Exclusion of Studies

After full-text evaluation, five studies were excluded because they did not give a quantitative analysis of bacterial resistance and six studies were excluded because they did not compare different antibiotic regimens.

### 2.3. Included Studies

The three studies were randomized controlled trials carried out in different countries: France, Sweden, and Brazil.

Two trials were conducted at University dental clinics [18,19] whereas one trial was conducted in a hospital [20]. Characteristics of the three included studies are reported in Table 1.

### 2.4. Characteristics of Participants

All studies included adult patients (age range: 18–81 years) [18,19,20]. Patients affected by *Helicobacter pylori* infection, chronic periodontitis, or whose treatment plan included at least one tooth extraction belonged to the test group. In the test group patients were prescribed an antibiotic for a determined period; in the control group patients were prescribed alternatively another antibiotic, the same antibiotic for a shorter amount of time, or placebo. Patients were excluded if they matched at least one of the following exclusion criteria: heart or renal failure, cancer, pregnant, nursing, any antibiotic molecule taken in the previous 45 days, history of allergic reaction to the tested antibiotic.

### 2.5. Characteristics of Interventions

Chardin et al. enrolled patients recruited from emergency dental consultations of three university hospitals in case they had an absolute indication for tooth extraction (destructive caries or traumatic, periodontal, and endodontic untreatable lesions). Patients were divided into two groups: the first group was given 1 g amoxicillin twice daily orally therapy for 3 days with a subsequent 4-day placebo period. The second group was given 1 g amoxicillin twice daily orally therapy for 7 days. The administration of amoxicillin started on day 0 (participant’s inclusion in the study). The oral surgery procedure was performed 2 days after the beginning of the antibiotic treatment (day 2); the follow-up period lasted for 7 days after tooth extraction (until reaching day 9). The authors also planned an additional follow-up period of around 1 month (until reaching day 30). Researchers evaluated the bacterial susceptibility to amoxicillin in each study group; such evaluation was performed analyzing the proportion of streptococci with reduced susceptibility out of their total number [20].

In the RCT of Feres et al., the authors evaluated the antibiotic resistance in subgingival plaque samples of patients affected by chronic periodontitis whose diagnosis had been made more than 5 years earlier. Such samples were taken in different time frames: baseline, at 3, 7, and 14 days during antibiotic administration and at 3, 7, 14, and 90 days post antibiotic therapy. All patients belonging to the test group were enrolled after a baseline-monitoring visit and before receiving a full mouth SRP under local anesthesia and instructions about proper home care procedures; in that occasion the authors took the first sample. Ten patients belonging to the test group were given orally metronidazole (250 mg, three times a day for 14 days) and 10 orally amoxicillin (500 mg, three times a day for 14 days). The antibiotic therapy started at the baseline-monitoring visit. At the end of the study period (day 90), researchers took a further sample from each patient, and everyone was given full mouth maintenance SRP. All samples were taken using sterile curettes from the distal aspects of six posterior teeth with pocket depth of at least 5 mm [18].

In the double blind RCT conducted by Stark et al. the authors enrolled 28 subjects that were positive for *Helicobacter pylori* infection by the rapid urease test and whose diagnosis had been confirmed by culture, histology, or PCR at least a year earlier. For the treatment of this condition 14 patients received 20 mg of omeprazole capsules (Losec, Astra, Södertälje, Sweden) and 1 g amoxicillin capsules (Amoxicillin Scand Pharm, Scand Pharm, Stockholm, Sweden) twice daily for 14 days (Group 1). Fourteen patients received omeprazole capsules 20 mg along with placebo (Apoteksbolaget, Stockholm, Sweden) capsules twice a day for the same period (Group 2). Eight men and six women composed the group 1; among them seven patients were affected by peptic ulcer disease and seven patients by dyspepsia without active ulceration. Patients belonging to group 2 included seven men and seven women; among them eight patients were affected by peptic ulcer disease and six by dyspepsia without active ulceration. No statistically significant differences were retrieved between the two groups regarding age, sex, or endoscopic diagnosis. Patients returned to the endoscopy ward on treatment day 10 and 28 days after treatment (day 42) for endoscopy and bacteriological sampling. Oral samplings were collected before gastroscopy with the aim of avoiding contamination with the gastric microflora [19].

### 2.6. Characteristics of Outcome Measures

All articles reported the percentage of bacterial cultures considered as resistant to the Minimum Inhibitory Concentration (MIC) of antibiotic necessary to inhibit the growth of 50% of its strains.

### 2.7. Risk of Bias in Included Studies and Strength of Evidence

The risk of bias is summarized in Table 2. Following the evaluation of the risk of bias for each study according to the Cochrane Collaboration Tool for six domains, two trials [18,19] were assessed as at high risk, whereas one as at medium risk [20].

### 2.8. Effects of Interventions

In the first 9 days Chardin et al. found an increase of more than 20% (from 1.3% to 23%) of the proportion of streptococci with amoxicillin reduced susceptibility in the first group, whereas a reduction to 7.7% was noted on day 30. A similar trend was noted for patients belonging to the second group: proportions ranged from 1.7% on day 0 to 24.7% on day 9 to 7% on day 30 [20].

Feres et al. demonstrated a rapid increase of antibiotic-resistant isolates during the study period in all groups. Such increase was not maintained over time, indeed after 90 days the values of antibiotic-resistant isolates returned around the baseline levels. A low percentage of amoxicillin resistant isolates was noted at the baseline (0.5%). A different baseline condition was noted regarding metronidazole since over 50% of isolates were found to be resistant before the molecule was administered. A rapid increase of the percentage of antibiotic-resistant isolates was noted in all groups during antibiotic administration and a slow decline after their withdrawal with an approximate return to baseline levels at 90 days [18].

Stark et al. only investigated resistance of enterobacteriaceae, arisen in fecal samples: analyzing the group 1 the authors reported that the MIC values had a significant increase during the treatment. Such values for enterococci and *E. coli* did not vary significantly comparing them in day 0 and day 42 although MIC values for enterobacteria other than *E. coli* increased permanently [19].

### 2.9. Antibiotic Working Principles

Since amoxicillin and metronidazole are the drugs used in the studies included in this review, it seems useful to underline their working principles.

**Figure 2:**
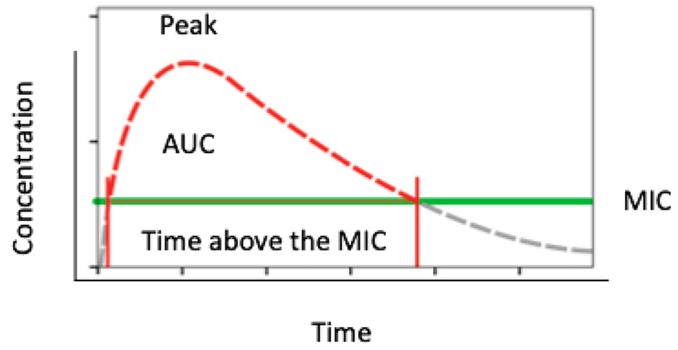
Figure showing pharmacodynamics of a time-dependent antibiotic.

**Figure 3:**
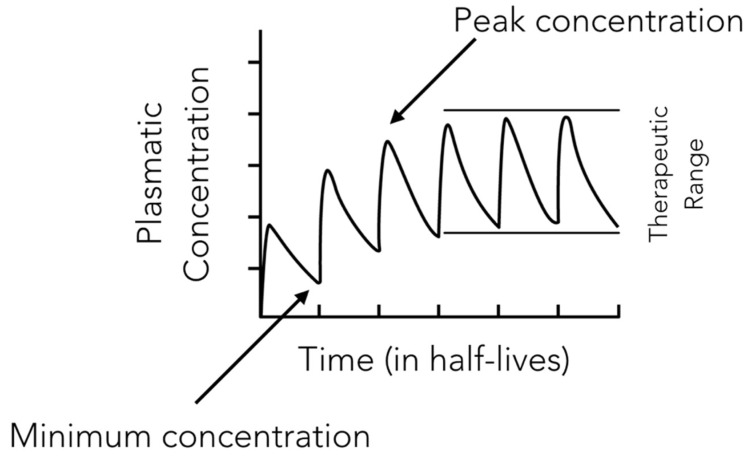
Figure showing how the therapeutic range is contained between the minimum and the peak concentration.

**Figure 4:**
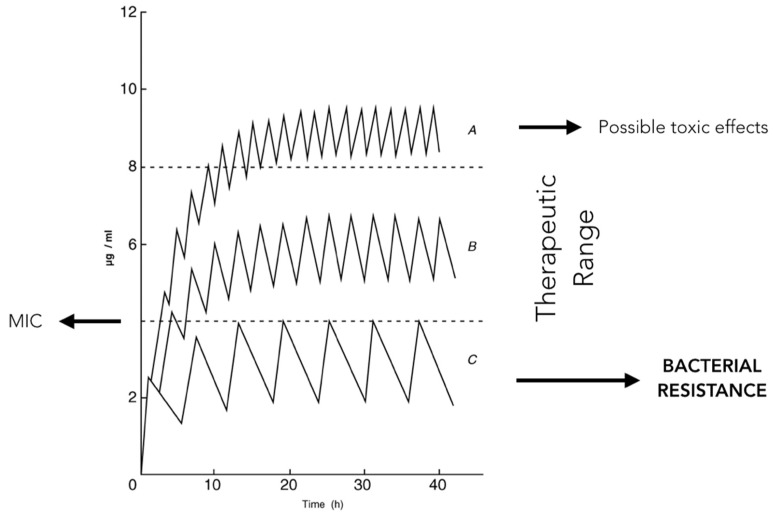
Figure showing how the modification of the interval of administration of a single dose can cause toxic effects or bacterial resistance.

Amoxicillin is a time-dependent antibiotic whose therapeutic action is closely related to the period of time during which the drug concentration is above the MIC (Figure 2). For this reason clinicians administering the drug should structure a posology aimed to maintain this parameter in the therapeutic interval for the time sufficient to totally inhibit the growth of the bacterial species responsible for the infection and to avoid possible toxic effects due to drug’s over-administration (Figure 3 and Figure 4).

Since amoxicillin has a half-life of approximately 90 min (but effective concentrations remain in circulation up to about 4–6 h), the dosing regimen that maintains the drug concentration above the MIC would be 500 mg every 6 h [21].

Metronidazole is a drug that is administered to manage anaerobic infections and, in the dental field, is considered off label in many countries: this is the reason why there are few trials to support its use in dentistry. Apart from that the dose administered by Feres (250 mg × 3 × 14 days) is, however, far below the effective dose use for proper applications (e.g., gynecology) [22].

It is hence clear that the inappropriate use of antibiotic molecules or intervals of administration lower than those required by the pharmacodynamics characteristics of the drug itself inevitably produces the failure to overcome the drug MIC and therefore the selection of resistant bacteria [1].

In light of drugs’ pharmacological properties, it is therefore not a viable behavior to reduce the number of days for antibiotic administration, but it is imperative that the drug is used within the correct dose intervals in the pre-established duration of the treatment [23].

## 3. Discussion

From the evidence drawn by the present review it could be demonstrated that a reduction of the amount of antibiotics or of its frequency of assumption does not influence the bacterial capacity to develop resistance. In fact, all the included articles reported a nonsignificant difference regarding the primary outcome of this review [18,19,20].

From the abovementioned information clinicians could be mistakenly induced to reduce the quantity or frequency of drug administration and prescription.

Nevertheless, it has to be pointed out that the studies included in this review used amoxicillin in concentrations and dosage regimen of 1 g every 12 h [19,20] or 500 mg every 8 h [18] and metronidazole with concentrations and dosage regimen of 250 mg every 8 h [18].

The choice of the dosage regimen seems to greatly depend on the habits of the clinician rather than on national or international guidelines. This aspect can be clearly understood from the great variability of prescription regimes for prophylaxis for oral surgery procedures reported in literature: the majority of studies opt for 2 g of amoxicillin before surgery [24,25,26] but also for other regimens as 2 g after surgery [27,28,29], 1 g twice a day for 7 days after surgery with or without 2 g before surgery [30,31,32] and for other combinations [33,34,35,36].

It could be simply gathered that the lack of adherence to guidelines is the main cause of the onset of bacterial resistance. Such statement, nevertheless, is in open contrast with the evidence that can be drawn from the results of the present review. On the one hand, in fact, the WHO reports a warning situation highlighting that current guidelines have not had the desired effect; on the other hand, the results found show that a change in the dosage regimens compared to the guidelines does not lead to an increase in the development of bacterial resistance.

In fact, some national guidelines are available, especially in Europe and North America, but several inconsistencies have been identified in the interpretation of evidence, and recommendations and validated systems to rank the evidence have seldom been used. Importantly, none of the currently available guidelines have been based on systematic reviews conducted ad hoc in order to provide evidence-based support for the development of recommendations. In addition, important topics with a global relevance that can lead to potentially harmful consequences for the patient if neglected are mentioned in only a few guidelines, for example, surgical hand antisepsis or the duration of surgical antibiotic prophylaxis (SAP) [37].

From the information reported in this review, therefore, it can be clearly understood that the most common antibiotics’ prescription regimens do not seem to be based on methodologically strong evidence [18,19,20]. Moreover, such regimens are, often, the result of experts’ opinions that could be related to the operator’s personal clinical experience in treating a particular disease [37].

The analyses carried out on oral bacterial flora and dental interventions have been predominant in the studies included in the revisions, so much so that a greater attention of the international dental scientific associations on this issue is needed.

In addition to what has been discussed so far it is mandatory to make some ethical and methodological considerations: despite the great number of studies retrieved by electronic databases only few studies investigated the target of this review. The reason for this evidence is that it is not ethical to compare short versus long periods of antibiotic treatment after any surgical intervention when the correct posology is already known. In fact, the WHO affirms that, in absence of infective complications, it is not ethical to prescribe antibiotics after every type of surgical intervention and that a scientific study that deals with an antibiotic regimen that goes against drug’s pharmacodynamics characteristics cannot be ethically admitted. This evidence is also underlined by the fact that the included studies usually do not contain the approval code of the ethics committee [19,20] or, in some cases, they do not even declare to have consulted it [18]. The general risk of bias, which was found to be medium-high (Table 2), also does not contribute to giving methodological consistency to the scientific evidence reported in the included studies.

Since it has been reported that in the daily clinical practice many dentists used to prescribe several days of antibiotics even after oral surgery and even without appropriate posology, it has to be pointed out that this malpractice is probably caused by the scarce knowledge of the WHO guidelines and of the pharmacological properties of the molecules [37]. Autonomy in interpreting the drug manufacturer’s recommendations may even be related to the operator’s personal clinical experience in treating a particular disease [38,39,40].

## 4. Materials and Methods

The present systematic review was written following the guidelines of the Preferred Reporting Items for Systematic Review and Meta-Analyses (PRISMA) [41].

A focused question, formulated in the PICO format (Patient, Intervention, Comparison, and Outcome), was developed: “Is the use of some antibiotics with different regimens responsible for the generation of microbial resistance in patients undergoing surgical interventions or in treatment for any type of infection?”

### 4.1. Protocol and Registration

The protocol for this systematic review was registered on PROSPERO (pre-registration code: CRD149149).

### 4.2. Eligibility Criteria

Population. Adult patients undergoing any type of surgical intervention or affected by any type of infection for which antibiotic therapy or prophylaxis was prescribed.

Intervention. Any type of antibiotic.

Comparison. Evaluation of different antibiotic regimens considered as the use of different molecules or as the use of the same molecules for different amounts of time.

Outcomes. Development of antibiotic resistance in single bacterial species or genus evaluated through cultural or metagenomic analysis.

Study design. All available randomized controlled trials (RCTs) and controlled clinical trials (CCTs).

In vitro or animal studies, studies dealing with subjects affected by systemic diseases, case reports, case series, review articles, abstracts, and discussions were excluded.

### 4.3. Information Sources and Search

An electronic search was conducted involving the following databases: MEDLINE-PubMed, Web of Science, Scopus, and the Cochrane Central Register of Controlled Trials (CENTRAL). No restrictions regarding language or publication date were applied. The searched was performed up to June 2019. The last search was conducted on 27 November 2019.

Databases were questioned with the following combination of MeSH terms and free text words: (((“Anti-Bacterial Agents/administration and dosage” [Mesh]) AND “Drug Administration Schedule” [Mesh]) OR (short course antibiotic AND long course antibiotic)) AND ((“Drug Resistance, Microbial” [Mesh]) OR antibiotic resistance). The abovementioned search strategy was the one used for Medline; the same strategy was, afterwards, adapted for the other databases. Gray literature was also screened questioning Google Scholar and considering the first 100 listed hits along with a supplementary manual search through Journal of Clinical Periodontology, Journal of Periodontology, Clinical Oral Implants Research, International Journal of Oral Implantology, International Journal of Oral and Maxillofacial Surgery, and International Journal of Oral and Maxillofacial Surgery. The manual search aimed at finding appropriate articles published up to November 2019. Lastly, all included articles were further analyzed checking their references with the aim of retrieving other relevant publications and contacting corresponding authors by e-mail for recovering unpublished articles or raw data.

### 4.4. Study Selection

Two reviewers (GM and LM) conducted the screening process independently and in duplicate. The screening process consisted of the evaluation of titles and abstracts of the retrieved studies according to the inclusion criteria. Subsequently, the same reviewers performed the assessment of the full-text articles. In case of disagreement the author supervisor (RP) was consulted.

### 4.5. Data Collection Process

The authors extracted the data with the help of data extraction forms specially designed for this purpose. In case of articles where the title and abstract information were not detailed enough in order to make a final decision, the reviewers screened the full-text article. Table 3 reports the list of the excluded studies after full-text evaluation, the reasons for exclusion are also provided.

### 4.6. Outcome

The primary outcome was the development of antibiotic resistance in single bacterial species or genus evaluated through cultural or metagenomic analysis. Summary measures were given as difference in means.

### 4.7. Risk of Bias in Individual Studies and Quality of Evidence

The reviewers independently extracted data regarding antibiotic resistance through a structured form.

The Cochrane Collaboration’s Risk of Bias tool was used for assessing risk of bias of all included RCTs and the Newcastle–Ottawa quality assessment scale for case control studies for all included CCTs, with the aim of defining their methodological quality.

### 4.8. Data Synthesis

The evaluation of the selected publications identified three studies [18,19,20]. All of them had considerable heterogeneity in terms of the targeted bacteria that prevented the authors from doing a quantitative analysis of data. For this reason, the authors decided to include all the three studies in the systematic review, but a meta-analysis was not performed.

## 5. Conclusions

The present review showed that different antibiotic regimens are not directly related to an increase in bacterial resistance against these molecules. This evidence, however, must be read in the light of a propensity to experiment with antibiotic doses other than those indicated by the manufacturers. This attitude has already been stigmatized by WHO which has also highlighted the ethical problems connected to it. Indeed, the pharmacokinetic and pharmacodynamics characteristics of the prescribed antibiotic molecules must be correctly known and considered when deciding to prescribe a therapy.

## Figures and Tables

**Table 1:** Characteristics of the included studies.

Title	Author (Year)	Type of Study	Sample Size	Mean Age (SD, Range)	Antibiotic Molecule and Regimen	Bacterial Resistance Tested
Reduced susceptibility to amoxicillin of oral streptococci following amoxicillin exposure	Chardin et al. (2009)	Randomized clinical trial	42 in test group; 39 in control group (no information about sex is given).	NR (NR, 19–45) for both groups.	Amoxicillin 1 g twice a day for 3 days and then placebo for test group. Amoxicillin 1 g twice a day for 7 days for control group.	Streptococci
Antibiotic resistance of subgingival species during and after antibiotic therapy	Feres et al. (2002)	Randomized clinical trial	10 (1 M and 9 F) in test group; 10 (6 M and 4 F) in control group	46 (15, NR) in test group; 42 (10, NR) in control group.	Amoxicillin 500 mg three times a day for 14 days for test group; metronidazole 250 mg three times a day for 14 days for test group.	Thirty-eight different species
Effects of omeprazole and amoxycillin on the human oral and gastrointestinal microflora in patients with *Helicobacter pylori* infection	Stark et al. (1996)	Randomized clinical trial	14 (8 M and 6 F) in test group; 14 (7 M and 7 F) in control group.	55.1 (NR, 22–78) in test group; 58.6 (NR, 26–81) in control group.	Amoxicillin 1 g and omeprazole 20 mg twice a day for 14 days for test group; omeprazole 20 mg twice a day for 14 days for control group	*Enterobacteriaceae* and *Escherichia coli*

M = male; F = female; SD = standard deviation; NR = not reported.

**Table 2:** Review of authors’ judgments on the sections of the Newcastle–Ottawa quality assessment scale for case control studies for each included study.

Title	Author (Year)	Random Sequence Generation	Allocation Concealment	Blinding of Participants and Personnel	Blinding of Outcome Assessor	Incomplete Outcome Data	Selective Reporting	Risk of Bias
Reduced susceptibility to amoxicillin of oral streptococci following amoxicillin exposure	Chardin et al. (2009)	1	1	1	0	1	1	Medium
Antibiotic resistance of subgingival species during and after antibiotic therapy	Feres et al. (2002)	1	0	1	0	1	1	High
Effects of omeprazole and amoxycillin on the human oral and gastrointestinal microflora in patients with *Helicobacter pylori* infection	Stark et al. (1996)	1	0	1	0	1	1	High

0 = Not satisfied; 1 = Satisfied.

**Table 3:** Table showing references of excluded studies after full text evaluation with rationale for exclusion.

Study	Rationale for Exclusion
Mathur et al.	No quantitative characterization of bacterial resistance
Kusachi et al.	No quantitative characterization of bacterial resistance
Avery et al.	No quantitative characterization of bacterial resistance
Her Young Su et al.	No quantitative characterization of bacterial resistance
Khariwala et al.	No quantitative characterization of bacterial resistance
Agarwal et al.	No comparison of different antibiotic regimens
Johnson et al.	No comparison of different antibiotic regimens
Liu et al.	No comparison of different antibiotic regimens
Luaces-Rey et al.	No comparison of different antibiotic regimens
McGowan et al.	No comparison of different antibiotic regimens
Rizk et al.	No comparison of different antibiotic regimens
